# Hepatitis B virus subgenotypes D1 and D3 are prevalent in Pakistan

**DOI:** 10.1186/1756-0500-2-1

**Published:** 2009-01-04

**Authors:** Saeeda Baig, Anwar Siddiqui, Runu Chakravarty, Tariq Moatter

**Affiliations:** 1 Department of Biochemistry, Ziauddin Medical College, Ziauddin University, Karachi, Pakistan; 2 Department of Biological and Biomedical Sciences, Juma Building, Aga Khan University, Karachi, Pakistan; 3 ICMR Virus Unit, ID & BG Hospital Campus, Kolkata, West Bengal, India; 4 Department of Molecular Pathology Juma Building, Aga Khan University, Karachi, Pakistan

## Abstract

**Background:**

As the hepatitis B genotyping is important for assessing its clinical implications and geographical distribution, the sub-genotypes have been found useful for determination of specific genomic markers related to hepatocarcinogenesis. In Pakistan, there is no reported data on molecular evolutionary analysis of HBV. A study was, therefore, much needed to evaluate the spectra of mutations present in the strains prevalent here.

**Findings:**

to confirm specificity of PCR typing, phylogenetic analysis of the pre-S1 region and the divergence was studied through 13 sequences of 362 bp (accession number EF432765 – EF432777). A total of 315 serum samples, selected from HBsAg positive patients representing the major ethnic groups, residing in Karachi, Sindh were tested for genotyping. Genotype D (219/315) was found to be the most prevalent (70%) amongst our patients. The rest of the genotypes A and a mixture of A and D (AD) were distributed as 20%, and 10% respectively. Phylogenetic tree demonstrated clustering of 11 samples with subgenotype D1 sequences and the remaining two strains on a branch within D3 samples. All samples intermixed with strains from other countries and were found to be closely related to Indian, Iranian and Egyptian HBV strains with 98.7 – 99.0% homology.

**Conclusion:**

This study confirms the predominance of genotype D in southeastern Asia and presence of subgenotypes DI and D3 in the Pakistani infected patients. More studies are required to investigate the reason for fewer inclusions of D3 compared to the D1 in Pakistani HBV strains.

## Findings

The structural and functional differences between HBV genotypes are the mainstay to severity, complications, treatment and possibly vaccination against the virus. Subgenotypes have been identified in different HBV genotypes on the basis of a > 4% (but < 8%) difference in the complete nucleotide sequences [1]. The phylogenetic method, a reliable tool to study the divergence in the HBV, are now being utilized to elucidate the history and origin of HBV genome scattered geographically around the world [2]. It has become well known that the genotypes/sub-genotypes vary in the geographical areas and correlate strongly with ethnicity [3]. Pakistan is one of the intermediate HBV endemic countries having distinct multiethnic populations. However, sufficient information on the molecular epidemiology of HBV is not available. There is no data on subgenotypes and their recombination or the phylogenetic relatedness of the virus endemic in the country. Karachi, the largest city of Pakistan and capital of the province of Sindh, is considered 'mini Pakistan' as all ethnic groups including Punjabis, Sindhis, Pukhtoons, Balochis and migrants from India and Afghanistan are living here. A study was therefore, undertaken to analyze DNA sequence of the 362 nucleotides of pre S gene of randomly selected HBV isolates from patients with hepatitis B in Karachi. The objective of the study, was to determine the genotypes and subgenotypes and to analyze the spectra of mutations through phylogenetic analysis of HBV strains prevalent in Pakistan.

## Results

### Prevalence of HBV/D as the most common genotype

Genotype D (219/315) was found to be the most prevalent (70%) amongst our patients. The rest of the genotypes A and a mixture of A and D (AD) were distributed as 20%, and 10% respectively (Table 1).

**Table 1:** Genotype distribution in the population studied.

**Genotype**	**Male** **(n = 237)**		**Female** **(n = 78)**		**Total** **(n = 315)**	
	
	**Number**	**Percent**	**Number**	**Percent**	**Number**	**Percent**
**A**	45	19	20	25	65	20

**D**	170	72	49	63	219	70

**AD**	22	9	9	12	31	10

### Dominance of subgenotype D1 and D3

**Figure 1:**
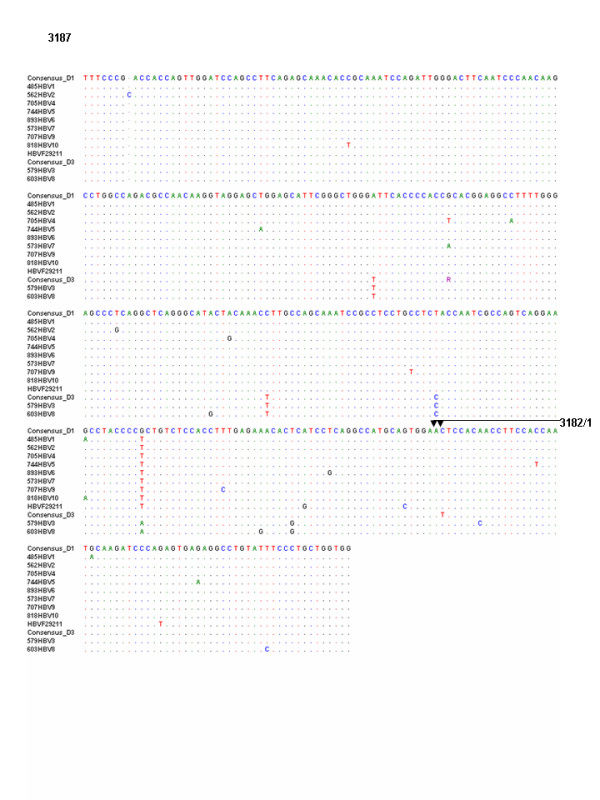
**The nucleotide sequences of the samples with genotype D were compared to those of reference D1 sequences **AY741798** from Iran, **AY161159** from India, **AY796032** from Turkey and reference D3 sequences **AY373430** from India, **EI00615** from Eastern India, **X85254** from Italy, **V01460** from France.**

**Figure 2:**
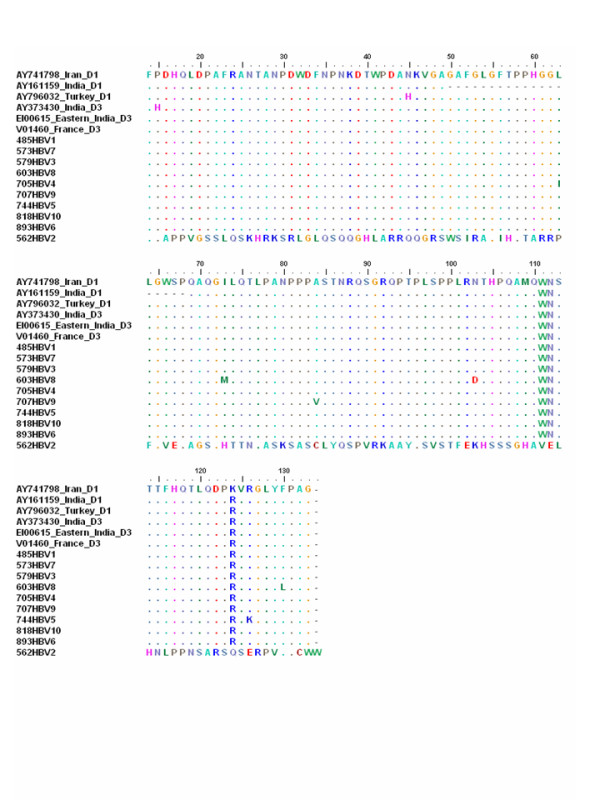
**Amino acid sequences (14–135) of part of the pre S protein for the HBV isolates from the study samples compared with the amino acid sequences of the same reference sequences**.

**Figure 3:**
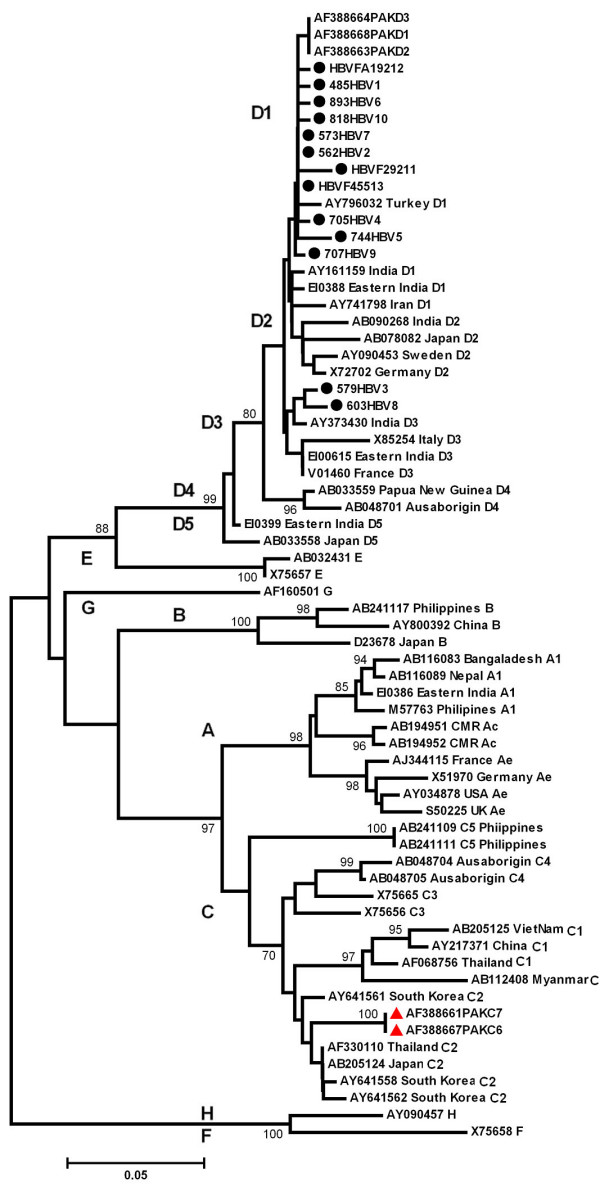
**Phylogenetic tree obtained by the neighbor-joining method**. Strains from this study are matched with strains obtained from GenBank indicated with their accession numbers. The designation and origin of the strains are given at the nodes of the branches.

The sequence of nucleotide and the amino acid residue are shown in Figures 1 and 2, respectively. Among the samples studied through phylogenetic analysis (Figure 3), all 13 belonged to genotype D, including 11 to subgenotype D1 and 2 to D3.

### Frequency and Distribution of Mutations in the PreS Gene

The nucleotide sequences of the samples with genotype D were compared to those of reference D1 sequences AY741798 from Iran, AY161159 from India, AY796032 from Turkey and reference D3 sequences AY373430 from India, EI00615 from Eastern India, X85254 from Italy and V01460 from France. In the 11 samples of genotype D, mutations were detected at 27 different sites and insertion of nucleotide C was noted after nt 2893 in sample 562HBV2 (Figure 1). Amino acid sequences of part of the pre S protein (14–135) for the HBV isolates from the study samples with genotype D were compared with the amino acid sequences of the same reference sequences (Figure 2).

## Discussion

On the basis of 362 nucleotides sequence in the pre S gene, this study demonstrates that Hepatitis B virus genotype D with subgenotype D1 and D3 is circulating in Karachi, Pakistan. Previously, several studies have been conducted which report Pakistan among many regions with intermediate to high HBV sero-prevalence, with D being the most prevalent genotype [4,5]. Our study was based on patients who, either were settlers in Karachi but originally belonged to different provinces or had come to Karachi for treatment. The pattern of genotype prevalence found through this study is in line with the studies from South East Asia, especially the countries sharing borders with Pakistan and thus confirms the high proportion of genotype D in southeastern Asia.

In Asia, research on genotyping, was initially conducted extensively in Japan and China, therefore, the genotypes of these countries namely B & C were considered as the most prevalent genotypes of Asia. Later on, when research was extended to other countries, predominance of D was found in South Asia and the Middle East such as India, Afghanistan and Iran [6-9]. The genotype D, in particular, happens to be most widely distributed and found universally, with its highest prevalence in a belt stretching from Southern Europe and North Africa to India, and in West and South Africa, having intravenous drug users as the most affected population within these countries [10,11]. This study, therefore, highlights the genotypic link between various ethnic groups within the country and people of the neighboring countries.

Phylogenetic analysis of genotype D could distinguish four subgenotypes, D1 to D5 [1,12]. The geographical distribution of the subgenotypes within D was found less restricted than that of genotypes A, B, and C, although the strains from Middle East mainly belonged to D1, those from South Africa and Alaska to D3, while those from Oceania and Somalia to D4. Whereas, D5 was only reported from Japan and India. The majority of the strains in this study belonged to subgenotype D1(84.6%). This subgenotype has mainly been shown in strains from Turkey, India, Iraq, Iran and Israel [11]. History of population migration from the Middle East to Pakistan is well known, which explains the predominance of HBV/D1 infection in this population. In neighboring India, genotype D is predominant with presence of subgenotypes D1, D2, D3 and D5 [1,12]. None of these subgenotypes was characterized by specific amino acid substitutions [11].

Two strains which clustered on a branch within the D3 samples EF432767 and EF584672 were more similar to each other than to the other D3 strains and seem to be derived from a phylogenetically related common ancestor. The *ayw3 *specifying strains within this subgenotype D3 have so far been reported from IDUs in Europe and the USA [11]. More studies are needed to investigate if the D3 strains have had fewer introductions into Pakistan compared to the D1 strains. The isolates studied, were found closely related to each other rather than with other D sequences, clustering near each other having 98.1–99% identity between the sequences. This close relationship demonstrates a consistent circulation of closely related viruses within this relatively multidimensional population.

Besides genotype D (76.3%), being the most prevalent, 20% of patients of the present study were found to be infected with genotype A, whereas 10% were co-infected with both types A and D. A recombination between genotypes A and D has also been reported from India [13]. Presence of mixed genotypes A and D in this study indicated a need for further studies for evaluation of possible recombination in samples from Pakistan. Studies from India have generally reported a similar pattern, predominance of genotype D coexisting with varying proportion of genotype A. HBV genotype D has also been described as the genotype of intravenous illicit drug users [14]. In a Pakistani study on IDUs by Alam et al. [5], the genotype distribution was found to be quite similar to the one in our study; genotype D at 62.5%; genotype A, 8.9% while 28.5% individuals were found to be infected with a mixture of genotype A & D. In our patients, the A and D combination was 10% compared to 28.5% reported earlier in Pakistani IDUs. This three times high percentage explains the cross contamination due to sharing of the syringes. Even triple infection has been reported in 0.9% of HBV infected intravenous drug users from areas where 3 HBV genotypes A, B and C are prevalent such as in China [15].

## Conclusion

This study confirms the predominance of genotype D in southeastern Asia. The sub-genotypes D1 and D3, dominate in the Pakistani patients. All isolates closely matched with those reported from Iran, India and Egypt. More studies are required to find out the genotypic heterogeneity as a result of intermixing with strains due to population migration.

## Methods

### Specimens

Blood was drawn from 350 patients undergoing treatment at Pakistan Medical Research Council (PMRC) and Ziauddin University Hospital (ZUH), diagnosed positive for HBV by HBsAg (ELISA – MUREX kit by Abbott Laboratories). The patients were randomly selected irrespective of their age and gender and a written consent (parental consent in case of under 18 years of age) was obtained. The study was approved by the Ziauddin University ethical review committee.

### DNA extraction and Amplification by PCR

DNA was extracted from 200 μl of serum using extraction kit (QIA AMP DNA mini kit 250 reactions Cat #51306). A modified version of nested PCR developed by Naito et al. [16] was followed for amplification. The step one PCR was carried out with universal outer primers P1 (sense) and S1-2 (antisense) in a tube containing 50 μl of a reaction buffer made up of the following components: 50 ng of each outer primer, a 200 μM concentration of each of the four deoxynucleotides, 1 U of Taq polymerase (Perkin-Elmer, Norwalk, Conn.), and 10× PCR buffer containing 1.5 mM MgCl_2_. The thermocycler (GeneAmp PCR system 2400, 9600, and 9700; Perkin-Elmer) was programmed to first incubate the samples for 2 min at 94°C, followed by 35 cycles consisting of 94°C for 20 s, 55°C for 20 s, and 72°C for 1 min with a final extension of 5 min at 72°C and incubation at 4°C. Two second-round PCRs were performed for each sample, with the common universal sense primer B2 used as the inner primer (sense) with a combination called mix A for genotypes A, B and C. Mix B consisted of antisense primer B2R with a combination of Mix B for genotypes D, E and F. A 2.5 μl aliquot of the first PCR product was added to two tubes containing the second sets of each of the inner primer pairs, each of the deoxynucleotides, Taq polymerase and PCR buffer, as in the first reaction and amplified for 35 cycles with: preheating at 94°C for 3 min, 15 cycles of amplification at 94°C for 20 s, 58°C for 30 s, and 72°C for 40 s, and an additional 20 cycles of 94°C for 30 s, 60°C for 30 s, and 72°C for 45 s with an extension of 7 min. at 72°C and incubation at 4°C. Genotypes of HBV for each sample were determined by identifying the genotype-specific DNA bands on electrophoresis. Positive PCR results were repeated twice for confirmation.

#### Sequencing

The PCR product of 1065 bp obtained with the universal primers P1 and S1-2 was sequenced in 13 HBV genotype D strains on an automated DNA sequencer by Gene Link USA.

#### Data analysis

Phylogenetic analysis was done using sequences from various countries retrieved from the international DNA database (DDBJ/EMBL/GenBank).

The best and the high scoring matches with our sequences were aligned with the CLUSTAL W software program [17] and the alignment was confirmed by visual inspection. Phylogenetic analysis of a 362 nt fragment of the S gene employing MEGA, version 2.0 [18], and PHYLIP, version 3.54c [19], was the basis for HBV genotyping using Kimura's 2-parameter algorithms with the neighbour-joining method. The reliability of different phylogenetic groupings was evaluated using the bootstrap test (1000 bootstrap replications). As almost identical groupings were observed with these tree-building programs, only the MEGA-based tree is presented.

The nucleotide sequence data reported in this paper has been submitted to the DDBJ/EMBL/GenBank databases and can be retrieved with accession number EF432765 through EF432777 at , EMBL in Europe and the DNA Data Bank of Japan.

ExPASy Translate tool, retrieved at  was used to translate the nucleotides (DNA) sequence of s protein to amino acid sequence. The deduced amino acid residues were sequentially set up in Figure 2.

## Competing interests

The authors declare that they have no competing interests.

## Authors' contributions

SB and AAS designed the Research project. SB carried out the molecular genetic studies, the sequence alignments and drafted the manuscript. TM and AAS helped in the bench work and optimization of the procedure. RC assisted in the Phylogenetic analysis of the sequences and helped to draft the manuscript. SB and RC wrote the final manuscript.
